# Socio-economic and environmental factors associated with high lymphatic filariasis morbidity prevalence distribution in Bangladesh

**DOI:** 10.1371/journal.pntd.0011457

**Published:** 2023-07-11

**Authors:** Tijana Williams, Mohammad Jahirul Karim, Shihab Uddin, Sharmin Jahan, Sultan Mahmood ASM, Shaun P. Forbes, Anna Hooper, Mark J. Taylor, Louise A. Kelly-Hope

**Affiliations:** 1 Centre for Neglected Tropical Diseases, Department of Tropical Disease Biology, Liverpool School of Tropical Medicine, Liverpool, United Kingdom; 2 Filariasis Elimination and STH Control Programme, Communicable Disease Control, Directorate General of Health Services, Ministry of Health and Family Welfare, Dhaka, Bangladesh; 3 Pedodontics Department, Bangabandhu Sheikh Mujib Medical University, Dhaka, Bangladesh; 4 Centre for Injury Prevention and Research, Dhaka, Bangladesh (CIPRB); 5 Center for Evidence Synthesis in Health, Brown University School of Public Health, Providence, Rhode Island, United States of America; 6 University of Nottingham, Nottingham, United Kingdom; 7 Institute of Infection, Veterinary and Ecological Sciences, University of Liverpool, Liverpool, United Kingdom; University of Liverpool, UNITED KINGDOM

## Abstract

**Background:**

Lymphatic filariasis (LF) is a vector-borne parasitic disease which affects 70 million people worldwide and causes life-long disabilities. In Bangladesh, there are an estimated 44,000 people suffering from clinical conditions such as lymphoedema and hydrocoele, with the greatest burden in the northern Rangpur division. To better understand the factors associated with this distribution, this study examined socio-economic and environmental factors at division, district, and sub-district levels.

**Methodology:**

A retrospective ecological study was conducted using key socio-economic (nutrition, poverty, employment, education, house infrastructure) and environmental (temperature, precipitation, elevation, waterway) factors. Characteristics at division level were summarised. Bivariate analysis using Spearman’s rank correlation coefficient was conducted at district and sub-district levels, and negative binomial regression analyses were conducted across high endemic sub-districts (n = 132). Maps were produced of high endemic sub-districts to visually illustrate the socio-economic and environmental factors found to be significant.

**Results:**

The highest proportion of rural population (86.8%), poverty (42.0%), tube well water (85.4%), and primary employment in agriculture (67.7%) was found in Rangpur division.

Spearman’s rank correlation coefficient at district and sub-district level show that LF morbidity prevalence was significantly (p<0.05) positively correlated with households without electricity (district r_s_ = 0.818; sub-district r_s_ = 0.559), households with tube well water (sub-district r_s_ = 0.291), households without toilet (district r_s_ = 0.504; sub-district r_s_ = 0.40), mean annual precipitation (district r_s_ = 0.695; sub-district r_s_ = 0.503), mean precipitation of wettest quarter (district r_s_ = 0.707; sub-district r_s_ = 0.528), and significantly negatively correlated with severely stunted children (district r_s_ = -0.723; sub-district r_s_ = -0.370), mean annual temperature (district r_s_ = -0.633.; sub-district r_s_ = 0.353) and mean temperature (wettest quarter) ((district r_s_ = -0.598; sub-district r_s_ = 0.316)

Negative binomial regression analyses at sub-district level found severely stunted children (p = <0.001), rural population (p = 0.002), poverty headcount (p = 0.001), primary employment in agriculture (p = 0.018), households without toilet (p = <0.001), households without electricity (p = 0.002) and mean temperature (wettest quarter) (p = 0.045) to be significant.

**Conclusions:**

This study highlights the value of using available data to identify key drivers associated with high LF morbidity prevalence, which may help national LF programmes better identify populations at risk and implement timely and targeted public health messages and intervention strategies.

## Author summary

Lymphatic Filariasis (LF) is particularly associated with poverty and living in a rural area, where disease transmitting mosquito vectors thrive. To better understand the socio-economic and environmental factors associated with the LF morbidity prevalence distribution in Bangladesh, this study examined publicly available data and used descriptive, statistical, and mapping methods to highlight key associations. Results found that high risk populations were those living in rural areas, employed in agriculture, with high levels of poverty, and houses without electricity or toilets, and where temperatures in the rainy season were lower than other regions of the country. These findings will help to inform public health messages and implement interventions for people affected by LF morbidity, but also to help reduce any current and future risk of transmission. This will support progress to achieving WHO elimination targets, leading to a future free of suffering for people affected by LF associated morbidity.

## Introduction

Lymphatic filariasis (LF) is a neglected tropical disease (NTD) that affects 70 million people worldwide [1]. It is caused by infection with the parasitic nematodes *Wuchereria bancrofti*, *Brugia malayi*, or *Brugia timori* and is transmitted through a variety of mosquito species [2,3]. LF is responsible for an estimated 2.78 million disability-adjusted-life years (DALYs) overall [4]. The disease manifests itself as disfiguring and painful lymphoedema, and/or hydrocoele that usually leads to permanent disability. The main aims of the Global Programme to Eliminate Lymphatic Filariasis (GPELF) are to interrupt transmission with Mass Drug Administration (MDA), and control suffering of patients with Morbidity Management and Disability Prevention (MMDP). Morbidity management is challenging and must be continued in endemic communities even after MDA has stopped, because affected patients remain in these communities for many decades [5].

Bangladesh was a highly endemic country that had made good progress since the inception of the Bangladesh LF Elimination Programme in 2000, and has recently achieved LF elimination as a public health problem [6,7]. In 2001, an estimated 70 million people were at risk across 34 endemic districts (19 high endemic–MDA required; 15 low endemic–no MDA required) with baseline infection rates between 1% and 15% caused by the parasite *W*. *bancrofti* and transmitted by the *Culex* spp. mosquitoes [8]. Over the past two decades, MDA and transmission assessment surveys (TAS) have been completed across 19 high endemic districts. MMDP activities have also been scaled up across all high and low endemic districts, including health worker training, facility assessments and surgeries, and patient searching [8]. The latter activity identified 44,410 LF clinical cases most of them in the 19 high endemic districts (98.4%), many of which were in the northern region of the country in Rangpur division, which is also one of the financially less developed [8].

The factors driving the high distribution of morbidity in the northern region of the country have not been examined but may be related to a combination of socio-economic and environmental characteristics that influence transmission. In Bangladesh, morbidity prevalence has been shown to correlate with baseline infection rates, i.e., transmission[8]. In the literature, several socio-economic factors have been associated with transmission including rural population, agricultural/outdoor employment, poverty, poor education, and poor housing infrastructure, such as the lack of good drainage, which may lead to ideal breeding conditions for *Culex* sp. mosquito vectors [9]. However, other factors that are proxies for poverty such as a lack of electricity (light in the night) [10], and nutritional status (e.g., malnutrition may affect ability of immunity to fight diseases) may also play a role [11–13].

The environmental factors that may influence transmission in Bangladesh are likely to be related to ecological requirements of the main mosquito species *Culex quinquefasciatus* and include a combination of climate and topographical characteristics [14]. Previous studies found that precipitation in the wettest quarter, population density and minimum temperature were important predictors of infection rates [14]. LF is particularly associated with living in a rural location, where there is presence of mosquito vectors transmitting the diseases [15–19]. In Bangladesh, *C*. *quinquefasciatus* breeds in polluted waters with organic material such as refuse, excreta and rotting plants [9,20–21].

The aim of this paper was to understand risk factors associated with the LF morbidity prevalence distribution and how this information may help to deliver interventions more efficiently for patients, by targeting areas with a high number of those affected. Specifically, key socio-economic and environmental risk factors were identified to help the programme implement appropriate interventions and reduce future risk. The Bangladesh LF programme has detailed national morbidity data and there are extensive data on socio-economic and environmental factors publicly available [22–25]. Morbidity prevalence estimates per 100,000 people were calculated using the population estimates based on the year of patient searching, which were extrapolated from the 2011 census using the annual growth rates. This provided the opportunity to examine the data collectively and identify key associations [8].

## Methods

### Study design

A retrospective ecological study was conducted to understand the association between LF morbidity prevalence and selected socio-economic and environmental risk factors in Bangladesh. Data were examined at division, district, and sub-district levels to assess disease and risk factor trends and associations across different geographical scales, which may help to develop and direct national, regional, and local intervention strategies.

### Study site

Bangladesh is a middle-income country in South-East Asia, with an estimated population of 168 million, and the highest population-density in the world [22]. The country has eight divisions (previously seven divisions until October 2021 when Mymensingh split from Dhaka division), 64 districts, 491 sub-districts (upazilas) and 87,310 villages [22]. Bangladesh is located on the coast of the sea of Bengal, it has a warm and humid climate, often affected by monsoons and heavy rains [26–27]. The landscape is diverse and includes coastal and marine ecosystems, terrestrial forests, hills, fresh waterways, and human settlements.

### Data sources

#### Morbidity data

LF morbidity data was based on the clinical cases identified during the LF programmatic patient searching activities between 2013–2016 and aligned with the administrative boundaries of the country at the time, as described in Karim et al. [8]. The number of cases ranged from 48 to 388,582 and prevalence rates from 1.5 to 280.9 per 100,000 population across the seven regional administrative divisions. Across the 34 LF endemic districts, the number of cases ranged from 0 to 11,199 (low endemic 5 to 138; high endemic 0 to 11,199) and prevalence rates from 0 to 568.9 per 100,000 population (low endemic 0.4 to 8.8; high endemic 0 to 568.9).

**Fig 1:**
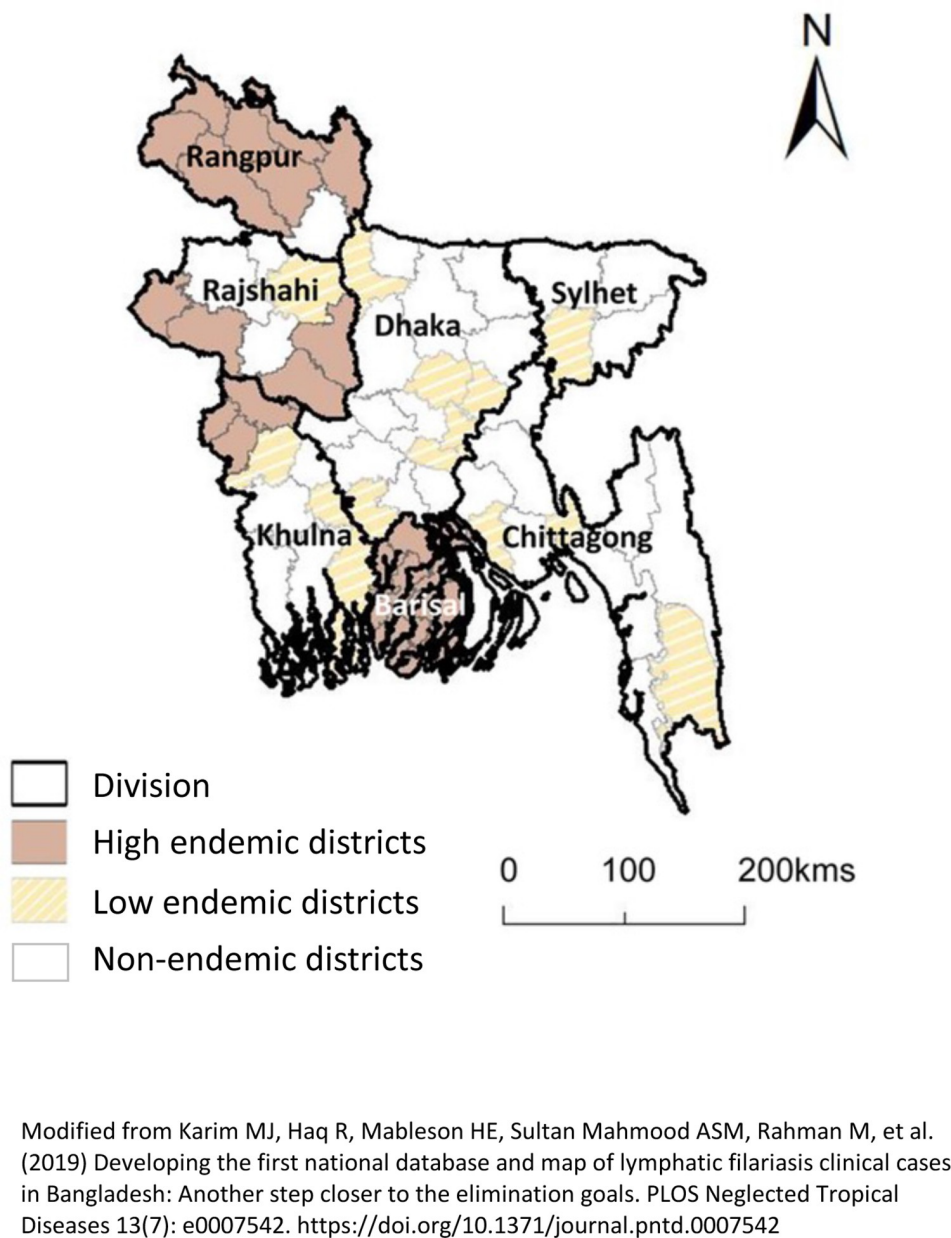
Bangladesh high and low endemic district by division. (adapted from Karim et al. [8].

This study specifically focussed on the variability found across the 19 high endemic districts. In total, 30,616 lymphoedema and 12,824 hydrocoele cases were examined together as ‘all conditions’ (n = 43, 678) and the related prevalence rate per 100,000 used in the analysis. The 19 high endemic districts comprise 132 sub-districts which broadly occur across three distinct geographical regions with widely different levels of morbidity. Fig 1 shows the distribution of the high and low endemic districts by division as presented previously in Karim et al. 2019 [8].

- the northern region has the highest LF burden and includes Rangpur division (LF cases n = 38,582; prevalence 280.9 per 100,000), seven districts (case range n = 1,958 to 11,199; prevalence range = 92.4 to 568.9 per 100,000), and 51 sub-districts (case range n = 0 to 2,724; prevalence range = 0 to 1,038 per 100,000)
- the central region has the lowest LF burden and includes two neighbouring divisions, Rajshahi and Khulna (LF cases n = 1,967; prevalence range 5.6 to 17.1 per 100,000), seven districts (case range n = 0 to 912, prevalence range = 0 to 53.4 per 100,000), and 59 sub-districts (case range n = 0 to 461; prevalence range = 0 to 84 per 100,000).
- the southern region has medium LF burden and includes the Barisal division (LF cases n = 3,129; prevalence 45.1 per 100,000), five districts (case range n = 351 to 1,023; prevalence range = 19.4 to 109.2 per 100,000), and 32 sub-districts (case range n = 4 to 368; prevalence range = 1 to 302 per 100,000)

#### Socio-economic data

Socio-economic data was obtained from the Bangladesh Interactive Poverty Maps which contain socioeconomic data at district and sub-district level available from the World Bank Group [23]. The data are a compilation of information from three different sources which are detailed in the primary data source and include data from 2010 Bangladesh Poverty Maps, the 2011 Census of Population and Housing sample available from the Integrated Public Use Microdata Series project (IPUMS), and the 2012 Undernutrition Maps of Bangladesh.

We included the factors identified in literature as those that may facilitate infection and/or transmission (Table 1) with the focus on the 19 high-endemic districts.These factors were grouped into five main categories including i) nutrition status ii) population/poverty iii) employment iv) education v) household infrastructure. Each factor was examined at district and sub-district level by comparative relation to prevalence.

**Table 1:** Summary of socio-economic and environmental factors examined by district and sub-district.

Theme	Socio-Economic Factor (measure)	Data source
**Nutrition status**	Severely underweight children (%)	World Bank Group (Poverty Maps) [23] (Data are based on three national sources between 2010–2012, with combined data and maps online in 2016.)
	Severely stunted children (%)	
**Population/Poverty**	Rural population (%)	
	Poverty head count (%)	
**Employment**	Primary employment in agriculture (%)	
**Education**	Primary education attainment (%)	
**Household infrastructure**	Households without electricity * (%)	
	Households with tube well water (%)	
	Households without toilet (open defecation) (%)	
**Theme**	**Environment Factor (measure)**	**Data source**
**Temperature**	Annual mean temperature (°C)	WorldClim [24] (Data are long term interpolated climate surface estimates (1970–2000), published in 2017 and version 2.1 online in 2020)
	Mean temperature of wettest quarter (°C)	
**Precipitation**	Annual precipitation (mm)	
	Precipitation of wettest quarter (mm)	
**Elevation**	Elevation (m)	NOAA GLOBE [27] (Data are derived from 11 sources to produce GLOBE Land One-kilometer Base Elevation, first published in 1999)
**Waterway**	Distance to nearest waterway (m)	WorldPop Hub [28] (Geospatial covariate data from distance to Open Street Map (OSM) major waterways 2016, Bangaldesh, published in 2018)

Note:

* Data were available on the households with electricity however for the purpose of this study the inverse was captured in results.

#### Environmental data

Environmental data was obtained from various global sources with information on climate and topographic features and include WorldClim temperature and precipitation surface estimates [24], National Oceanic and Atmospheric Administration (NOAA) Global Land One-km Base Elevation Project (GLOBE) [27] and WorldPop Hub distance to OSM (Open Street Map) major waterways [28]. We included the factors identified in literature as those that may facilitate infection and/or transmission (Table 1) with the focus on the 19 high-endemic districts. These factors were grouped into four main categories i) temperature (degrees Celsius,°C) ii) precipitation (millimetres, mm), iii) elevation (metres, m), vi) distance to nearest major waterway, from the cell centre to the nearest feature/river (metres, m).

The climate, elevation and waterway data were raster-based and information extracted to the administrative boundaries available from Geo-DASH (https://geodash.gov.bd) using the geoprocessing spatial tool in mapping software ArcGIS 10.8.1 (ESRI, Redlands, Ca). Each factor was examined at district and sub-district level by the mean and standard deviation of the specific measure.

### Data analysis and mapping

Data were compiled into an Excel file (Microsoft) [29] and imported into the ‘R’ statistical analysis software [30] for descriptive and statistical analysis at division, district, and sub-district levels. District data are available in S1 Table and sub-district data in S2 Table.

First, the socio-economic and environmental factors were summarised for each administrative division (n = 7) with the highest and lowest measures highlighted. In addition, the socio-economic and environmental factors at district and sub-district levels were summarised with the mean, standard deviation and range tabulated.

Second, bivariate analysis was conducted to identify the factors most significantly correlated with LF morbidity prevalence in the high endemic districts (n = 19) and sub-districts (n = 132). The Spearman’s correlation coefficient was used and the correlation direction (i.e., positive, or negative) and statistical significance (2-tailed, p value <0.05) were tabulated and summarised. For the risk factor relating to households *with* electricity, the inverse *without* electricity was used in final results.

Third, negative binomial regression analysis was conducted to identify the factors, such as the proportion of houses without electricity (independent variable), that were associated with the total cases of LF (lymphoedema and hydrocoele)(dependent variable) in the high endemic districts at sub-district level (n = 132). The total population for each sub-district was used as an offset on the log scale. Negative binomial regression analysis was conducted using LF case data and key socio-economic and environmental factors. To account for factors that were highly correlated (r ≥ 0.8) with each other, i.e., reduce collinearity, Spearman’s correlation was conducted between them. In the analysis, the level of collinearity tolerance was set at ≥0.8 and for pairs of variables with correlation above this tolerance, one of the variables was excluded from the regression model. Coefficients between 0 and 1 indicated a negative association and above 1 a positive association with statistical significance (p-value ≤0.05). The MASS package in R was used to estimate the negative binomial regression model.

Finally, to visually highlight the distribution of socio-economic and environmental factors across the endemic areas, maps were produced in ArcGIS 10.8.1 (ESRI, Redlands, Ca) using administrative boundaries available from Geo-DASH (https://geodash.gov.bd). The factors were grouped into four categories and defined using the Jenks Natural Breaks algorithm to help minimize the variation within each range. The maps created for socio-economic and environmental factors therefore had unequal values presented in the scale.

### Ethics statement

The LF morbidity data were collected as part of routine programme activities conducted by the Ministry of Health and Family Welfare (MOHFW), Bangladesh, and therefore no ethical clearance was required for secondary data analysis. Ethical approval was obtained from the Liverpool School of Tropical Medicine Research Ethics Committee (Research Protocol 12.22) to support MOHFW programme activities within the initial case finding activities and the subsequent analysis of data. All data were anonymised.

## Results

### Division summaries

Division level data for LF morbidity prevalence, socio-economic and environmental factors are presented in Table 2. For socio-economic factors, Rangpur division was found to have the highest rural population (86.8%), poverty head count (42.0%), households with tube well water (85.4%) and primary employment in agriculture (67.7%). Sylhet division was found to have the highest rates of severely underweight (9.6%) and severely stunted (29.8%) children.

**Table 2:** Summary of LF morbidity prevalence, socio-economic and environmental factors by division.

		Rangpur	Rajshahi	Khulna	Barisal	Dhaka	Chittagong	Sylhet
**LF morbidity prevalence**	Per 100,000	280.9*	17.1	5.3	45.1	1.5†	3.7	2.2
**Socio-Economic Factors**		**%**	**%**	**%**	**%**	**%**	**%**	**%**
**Nutrition**	Severely underweight children (%)	7.8	6.6	5.3†	6.9	5.6	6.5	9.6*
	Severely stunted children (%)	18.7	19.8	16.6†	20.9	18.6	16.9	29.8*
**Population**	Rural population (%)	86.8*	82.3	82.2	83.9	67.2†	75.9	85.3
	Poverty head count (%)	42.0*	27.4	32.0	38.4	30.2	25.8	25.0†
**Employment**	Primary employment in agriculture (%)	67.7*	62.6	58.4	58.4	36.5†	39.6	54.3
**Education**	Primary education attainment (%)	30.6	36.0	45.2	45.3	42.2	58.7*	29.2†
**Household**	Households without electricity (%)	71.2	57.9	66.0	34.0†	65.1	91.7*	50.0
	Households with tube well water (%)	85.4*	72.9	53.9	75.2	41.0	24.4	77.3
	Households without toilet (%)	20.3	11.5	4.3	75.2*	5.2	4.1 †	8.5
**Environmental Factors**		**Mean**	**Mean**	**Mean**	**Mean**	**Mean**	**Mean**	**Mean**
**Temperature**	Mean temperature (wettest quarter) (°C)	28.5	28.9	29.1*	28.5	28.5	27.5 †	28.0
	Mean annual temperature (°C)	24.9 †	25.5	26.0*	25.9	25.5	25.3	24.9 †
**Precipitation**	Mean annual precipitation (mm)	2,386.0	1,584.7 †	1,681.5	2,378.3	2,106.8	2,995.9*	2,489.9
	Mean precipitation of wettest quarter (mm)	1,457.1	891.7 †	960.2	1,409.5	1,113.1	1,916.5*	1,273.8
**Elevation**	Mean elevation (m)	38.2	17.5	11.9	1.8 †	6.9	60.9*	8.9
**Waterway**	Mean distance to nearest waterway (m)	3.2	5.9	5.8	5.5	3.1 †	6.6*	6.1
**Key**	Highest	*						
	Lowest	†						

For environmental factors, Chittagong division was found to have the highest mean precipitation (2995.9mm), mean precipitation of wettest quarter (1916.5mm), elevation (60.9m), distance to nearest waterways (6.6m), and Khulna division the highest mean temperature (29.1°C) and annual temperature (26.0°C). Rangpur division was found to have the lowest mean temperature of wettest quarter (24.9°C). The main landcover category across all divisions was irrigated croplands, however, the Khulna division and Chittagong division has additional landcover types.

### District and sub-district summaries

The LF morbidity prevalence, socio-economic and environmental summary measures (mean, standard deviation, and range) by district and sub-district varied widely as shown in Table 3. The socio-economic factors with the highest mean measures included households with tube well water (district 93.1%, 5.5 SD, 74.6–97.6%; sub-district 93.3%, 8.4 SD, 37.4–98.9% range) and the lowest mean measures included the severely underweight children (district 7.8%, 0.9 SD, 6.1–9.6%; sub-district 7.8%, 1.2 SD, 4.0–10.6% range). The environmental measures were similar at district and sub-district levels with mean temperature measures ranging between 25.5–28.7°C, precipitation measures 1197.0–2068.4mm, elevation 16.0–20.3m and distance to nearest waterway 4.5–4.7m.

**Table 3:** Summary of LF morbidity prevalence, socio-economic and environmental factors by overall districts and sub-districts.

		District Level			Sub-district Level		
		Mean	SD	Range	Mean	SD	Range
LF morbidity prevalence per 100,000 population		133.7	170.0	0–568.9	123.3	196.25	0–1038.7
**Socio-Economic Factors**							
**Nutrition**	Severely underweight children (%)	7.8	0.9	6.1–9.6	7.8	1.2	4.0–10.6
	Severely stunted children (%)	21.9	2.2	19.0–26.8	21.6	2.4	16.7–30.6
**Population**	Rural population (%)	84.0	5.7	67.8–90.5	82.9	17.8	0.0–100
	Poverty head count (%)	33.1	13.8	3.6–63.7	35.4	14.2	3.0–68.8
**Employment**	Primary employment in agriculture (%)	62.2	7.7	47.5–72.9	62.8	17.0	4.8–86.9
**Education**	Primary education attainment (%)	33.1	6.6	24.8–46.0	32.8	7.2	19.4–49.3
**Household**	Households without electricity (%)	43.5	14.3	20.2–64.3	43.6	17.7	12.3–95.2
	Households with tube well water (%)	93.1	5.5	74.6–97.6	93.3	8.4	37.4–98.9
	Households without toilet (%)	9.9	8.2	1.3–27.0	10.0	9.7	0.2–48.9
**Environmental Factors**							
**Temperature**	Mean temperature (wettest quarter) (°C)	28.7	0.33	28.2–29.3	28.7	0.32	27.8–29.4
	Mean annual temperature (°C)	25.5	0.49	24.8–26.1	25.5	0.43	24.7–26.1
**Precipitation**	Mean annual precipitation (mm)	2068.4	477.3	1352.2–2747.5	2043.0	425.9	1317.8–3072.8
	Mean precipitation of wettest quarter (mm)	1229.4	318.3	808.3–1697.3	1197.0	282.9	797.1–1896.8
**Elevation**	Mean elevation (m)	20.3	16.23	1.0–57.3	16.0	19.6	0.0–139.4
**Waterway**	Mean distance to nearest waterway (m)	4.5	1.82	2.5–8.7	4.7	2.8	1.0–15.7

### Bivariate analysis

Bivariate correlations between LF morbidity prevalence and the socio-economic and environmental factors at district and sub-district level are shown in Table 4.

**Table 4:** Spearman’s rank correlation between LF morbidity prevalence, socio-economic and environmental factors and at district and sub-district level.

Socio-Economic		District level 19 high endemic		Sub-district level 132 high endemic	
Group	Indicators–ALL	Coefficient++	P-Value	Coefficient++	P-Value
**Nutrition**	Severely underweight children (%)	0.309	0.198	0.201	0.021*
	Severely stunted children(%)	-0.723	<0.001*	-0.370	<0.001*
**Population**	Rural population (%)	0.374	0.116	0.316	<0.001*
	Poverty head count (%)	0.247	0.306	0.082	0.349
**Employment**	Primary employment in agriculture (%)	0.377	0.112	0.183	0.036*
**Education**	Primary education attainment (%)	-0.140	0.565	-0.110	0.207
**Household**	Households without electricity (%)	0.818	<0.001*	0.559	<0.001*
	Households with tube well water (%)	0.400	0.091	0.291	<0.001*
	Households without toilet (%)	0.504	0.030*	0.400	<0.001*
**Environment**			
**Group**	**Indicators**	**Coefficient++**	**P-Value**	**Coefficient++**	**P-Value**
**Temperature**	Mean temperature (wettest quarter) (°C)	-0.598	0.008*	-0.316	<0.001*
	Mean annual temperature (°C)	-0.633	0.004*	-0.353	<0.001*
**Waterways**	Distance to nearest waterway (m)	-0.414	0.079	-0.166	0.057
**Precipitation**	Mean annual precipitation (mm)	0.695	0.001*	0.503	<0.001*
	Mean precipitation of wettest quarter (mm)	0.707	0.001*	0.528	<0.001*
**Elevation**	Mean elevation (m)	0.558	0.015	0.000	0.995

++ Spearman’s rank correlation coefficient rho (r_s_)

* Significant P-value ≤0.05

For socio-economic factors, LF morbidity prevalence at both district and sub-district level was significantly (p<0.05) positively correlated with households without electricity (district r_s_ = 0.818; sub-district r_s_ = 0.559), households with tube well water (sub-district r_s_ = 0.291), and households without toilet (district r_s_ = 0.504; sub-district r_s_ = 0.40), and significantly negatively correlated with severely stunted children (district r_s_ = -0.723; sub-district r_s_ = -0.370). Additional significant positive correlations at sub-district level were found with severely underweight children (r_s_ = 0.201), rural population (r_s_ = 0.316) and employment in agriculture r_s_ = 0.183).

For environmental factors, LF morbidity prevalence at both district and sub-district level was significantly (p<0.05) positively correlated with mean annual precipitation (district r_s_ = 0.695; sub-district r_s_ = 0.503), mean precipitation of wettest quarter (district r_s_ = 0.707; sub-district r_s_ = 0.528), and significantly negatively correlated with mean annual temperature (district r_s_ = -0.633.; sub-district r_s_ = 0.353), and mean temperature during wettest quarter (district r_s_ = -0.598; sub-district r_s_ = -0.316).

### Negative binomial regression analysis

The sub-district level regression results are presented in Table 5. The number of LF morbidity cases was found to be significantly associated with severely stunted children (p = <0.001), rural population (p = 0.002), poverty headcount (p = 0.001), primary employment in agriculture (p = 0.020), households without toilet (p = <0.001), households without electricity (p = 0.002) and mean temperature (wettest quarter) (p = 0.045). Mean annual precipitation was excluded from the analysis as it correlated with mean precipitation (wettest quarter) with a correlation coefficient ≥0.8.

**Table 5:** Negative binomial regression results for LF morbidity cases, and associated socio-economic and environmental factors at sub-district level.

Group	Indicators	Exponentiated Coefficient	P-Value
**Nutrition**	Severely underweight children (%)	6148942	0.594
	Severely stunted children (%)	0.000	<0.001*
**Population**	Rural population (%)	53.008	0.002*
	Proportion of poverty headcount (%)	0.037	0.001*
**Employment**	Primary employment in agriculture (%)	0.031	0.018*
**Education**	Primary education attainment (%)	1.996	0.835
**Household**	Household without toilet (%)	362.235	<0.001*
	Households with tube well water (%)	1.082	0.955
	Households without electricity (%)	53.824	0.002*
**Temperature**	Mean annual temperature (°C)	0.904	0.798
	Mean temperature (wettest quarter) (°C)	0.254	0.045*
**Precipitation**	Mean precipitation of wettest quarter (mm)	1.000	0.612
**Elevation**	Mean Elevation (m)	1.002	0.719
**Waterways**	Distance from nearest waterway (m)	0.952	0.233

* Significant P-value ≤0.05

^ Estimates <1 indicate negative associations and >1 positive associations

### Sub-district level maps

**Fig 2:**
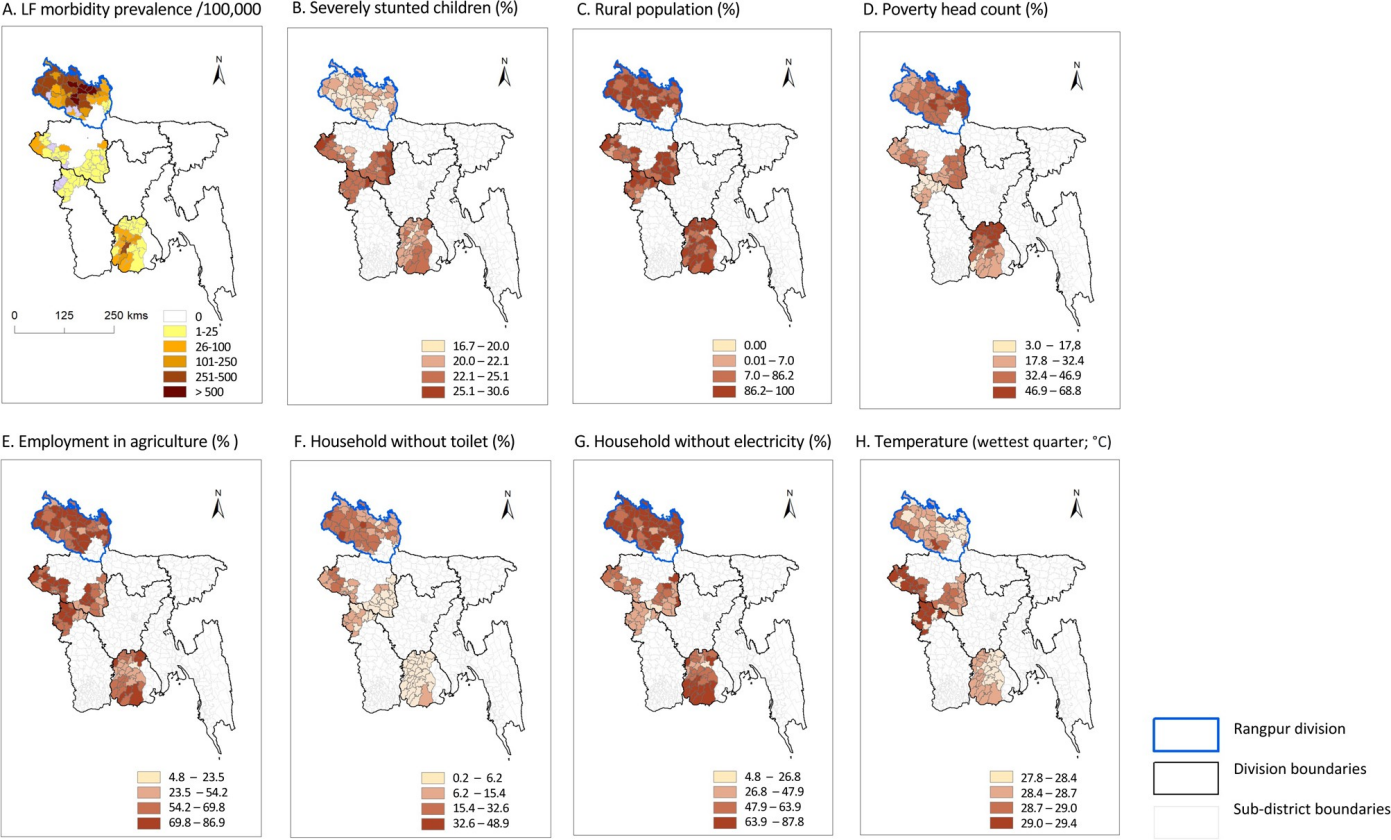
Maps of LF morbidity prevalence and significant socio-economic and environmental factors at sub-district level. Note: the scales vary for each map as the divisions are based on natural jenks.

The sub-district level maps of LF morbidity prevalence, and the socio-economic and environmental factors found to be significant in the binomal regression analysis are shown in Fig 2A–2H. The maps highlight in Rangpur division that the distribution of severely stunted children (Fig 2B) (16.7–22.1% range) is overall low, whereas rural populations (0–100%), employment in agriculture (23.5–86.9%) and households without electricity (34.8–95.2%) and mean temperature (wettest quarter; 6.2–48.9) are predominately higher than in other divisions and regions of the country.

## Discussion

This study highlights how the use of publicly available socio-economic and environmental data may be used to better understand the factors associated with high LF morbidity prevalence in an endemic country. The examination of data at different geographical scales also demonstrated how the broader regional trends may provide insights into specific risk factors at a local level. For example, the highly LF endemic Rangpur division had the highest regional number of people living in rural areas, in poverty and primarily employed in agriculture, which were also found to be significantly associated with LF morbidity prevalence at the sub-district level together with additional factors.

The risks associated with socio-economic and environmental factors are important to identify, as many agricultural workers living in poor rural areas may be more exposed to infectious mosquito bites, leading them to being disproportionately afflicted by clinical conditions [8,18]. Furthermore, populations living in poverty often have inadequate housing infrastructure, and water and sanitation facilities, including toilet and drainage systems [23]. This combination of factors was found to be significant in our regression analysis, and can indirectly support favourable breeding grounds for the *Culex* mosquito vectors, which thrive in polluted waters with organic material such as refuse, excreta and rotting plants [9,20–21]. Recent suitable breeding sites found in LF endemic areas of Rangpur division include water pits, septic tanks, blocked drains, canals, and abandoned wells [9]

In this paper we included both socio-economic and environmental risk factors, which are not often considered together, with only a few examples related to infection [14,31–32]. While our district and sub-district correlation analyses showed important associations for both groups of risk factors, the regression analysis indicated that the socio-economic factors are the most prominent. These factors are proxies for poverty and social inequalities and have been shown to be important drivers of several NTDs [33]. Very few studies have examined these factors in relation to LF, however in neighbouring India, studies have found higher risks of infection in populations living in poor rural areas compared with poor urban areas [34] and in populations where agriculture was the main occupation, incomes were the lowest, drainage systems were open, and house construction lowly (i.e. tiled or hut type compared with concrete) [34–35].

LF morbidity prevalence is significantly negatively correlated with severely stunted children, which is somewhat counterintuitive as previous studies have indicated that poor nutrition may result in compromised immune system, and leave people vulnerable to infection, especially children [11–13]. The higher rates of LF morbidity occur in a largely agricultural areas, which may provide sufficient food for the communities that live there and reduce the risk of nutritional deficiencies and associated childhood stunting. The sub-district maps shown in this study highlight the lack of geographical overlap between high LF morbidity and severely stunted children, which further helps to explain the statistical results.

The environmental factors found to be correlated with high LF morbidity prevalence at district and sub-district level included the annual and seasonal temperature and precipitation measures. These climatic factors are key for maintaining vector transmission cycles and support findings from other studies in the region and elsewhere [14,20,36–37]. Irish et al. [9] noted that areas in the Rangpur division had favourable climate conditions for filariasis transmission for 4 to 8 months with moderate to high level of endemicity and rates of clinical disease. Rangpur division has the lowest regional mean annual temperature of 24.9°C and our finer scale analysis suggests that the lower temperatures are contributing factors, which may favour the *Culex* mosquitoes, as extreme heat or humidity may impair their ability to breed and/or transmit disease [38–39].

This study is the first to examine socio-economic and environmental risk factors associated with LF morbidity prevalence across an entire country and was only made possible by the extensive data collected by the Bangladesh LF programme [8]. However, there are several limitations that are important to address. The morbidity and risk factor data were collected at different time frames which limits the temporal comparability. It may have been beneficial to include LF infection rates in the analysis, however there was a lack of available data across the different geographical scales to conduct the analysis. Summarising data to district or sub-district levels averages out across a geographic scale and could misinterpret aspects of disease that are focal or make links between unrelated or by simple coincidence between factors and LF morbidity. The ranges of percentage captured in the sub-district level maps could be inaccurate, and the complete range corresponding to all divisions could be analysed. No spatial components were included in the models. It was also not possible to include any reference to future risk and is acknowledged that some of these risk factors will change and/or become obsolete over time. Further, the use of continuous variables may be programmatically difficult to interpret and can imply a greater relationship than exists when the underlying data only has minimal difference. Finally, the methodological approaches can only be generalised to countries that have such detailed morbidity data.

Notwithstanding these limitations, there are several strengths to this study, which add value to current knowledge gaps and are still relevant to the Bangladesh LF programme in the elimination phase. This study shows how national LF morbidity data may be used together with other data to identify key risk factors in high burdened areas, especially those related to socio-economic inequalities. This may help the Bangladesh programme consider future risk by monitoring demographic and ecological changes over time. The findings may be relevant and provide modelling opportunities to other endemic countries or areas in the South-East Asian region that have less morbidity data but similar socio-economic and environmental characteristics. Sabesan et al. (2012) found that creating a geo-environmental risk model to identify LF transmission risk areas in India was essential for development of targeted interventions and subsequent surveillance protocols [40].

Understanding the range of risk factors across the different geographical scales will help national and local programmes to target risk areas (e.g., poor rural agricultural areas) with appropriate morbidity and transmission related public health messages and interventions (e.g., vector control, environmental modification, and/or personal protection). Finally, the factors identified here for LF morbidity may be relevant to future LF transmission potential in areas at risk of ongoing transmission and recrudescence. This, therefore, will help to direct post-elimination surveillance strategies.

## Supporting information

S1 STROBE Checklist STROBE Statement for ecological study. (DOCX)

S1 Table District level LF morbidity prevalence, socio-economic and environmental data. (XLSX)

S2 Table Sub-district level socio-economic and environmental data. (XLSX)

S1 Fig Histogram of total prevalence per 100,000 people. (PDF)
