# Supplementary figures and images for: Socio-economic and environmental factors associated with high lymphatic filariasis morbidity prevalence distribution in Bangladesh
Source: PLoS Negl Trop Dis. 2023 Jul 11;17(7):e0011457. doi: 10.1371/journal.pntd.0011457 (PMC10335661; doi:10.1371/journal.pntd.0011457)

**S1 Figure. Total Prevalence per 100,000 people**

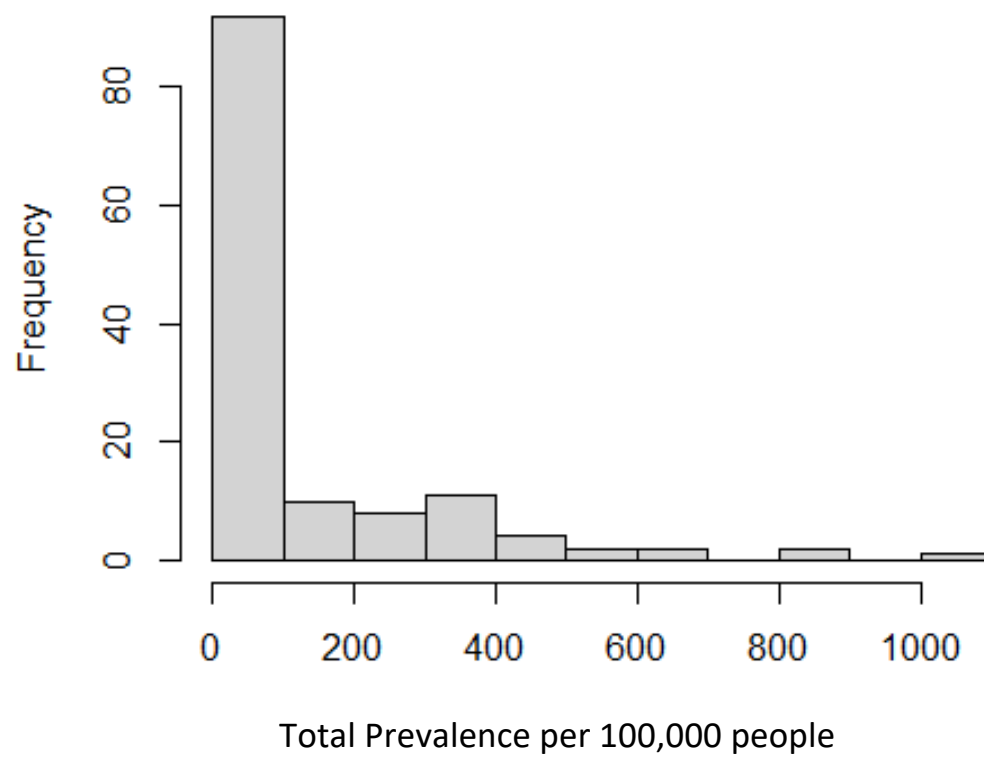

Supplement: S1 Fig — (PDF) [file pntd.0011457.s004.pdf]
